# The interplay of restriction-modification systems with mobile genetic elements and their prokaryotic hosts

**DOI:** 10.1093/nar/gku734

**Published:** 2014-08-12

**Authors:** Pedro H. Oliveira, Marie Touchon, Eduardo P.C. Rocha

**Affiliations:** 1 Institut Pasteur, Microbial Evolutionary Genomics, Département Génomes et Génétique, Paris, France; 2 CNRS, UMR3525, Paris, France

## Abstract

The roles of restriction-modification (R-M) systems in providing immunity against horizontal gene transfer (HGT) and in stabilizing mobile genetic elements (MGEs) have been much debated. However, few studies have precisely addressed the distribution of these systems in light of HGT, its mechanisms and its vectors. We analyzed the distribution of R-M systems in 2261 prokaryote genomes and found their frequency to be strongly dependent on the presence of MGEs, CRISPR-Cas systems, integrons and natural transformation. Yet R-M systems are rare in plasmids, in prophages and nearly absent from other phages. Their abundance depends on genome size for small genomes where it relates with HGT but saturates at two occurrences per genome. Chromosomal R-M systems might evolve under cycles of purifying and relaxed selection, where sequence conservation depends on the biochemical activity and complexity of the system and total gene loss is frequent. Surprisingly, analysis of 43 pan-genomes suggests that solitary R-M genes rarely arise from the degradation of R-M systems. Solitary genes are transferred by large MGEs, whereas complete systems are more frequently transferred autonomously or in small MGEs. Our results suggest means of testing the roles for R-M systems and their associations with MGEs.

## INTRODUCTION

The flow of genetic information between bacterial cells by horizontal gene transfer (HGT) drives bacterial evolution (1,2) and restriction-modification (R-M) systems are key moderators of this process (3,4). They are thought to be ubiquitous in bacteria and archaea (5), and operate like many poison-antidote systems: they typically encode a methyltransferase (MTase) function that modifies a particular sequence and a restriction endonuclease (REase) function that cleaves a DNA when its recognition sequence is unmethylated (6–8). The three classical types of R-M systems differ in their molecular structure, sequence recognition, cleavage position and cofactor requirements (9) (Supplementary Figure S1). Type I systems are complex hetero-oligomers either comprising one DNA sequence specificity (S), two REase and two MTase subunits with restriction and modification activities, or two MTase and one S subunits with modification activity only. Type II systems encoded on separate genes are composed of one homodimeric or homotetrameric REase and one monomeric MTase, and in most cases are able to operate separately and independently from each other at least *in vitro*. Some Type II systems, particularly Types IIB, IIG, IIL, and some IIH (collectively termed IIC) encode both restriction and modification domains within the same protein (10,11). Type III systems are heterotrimers or heterotetramers of products of two genes, *res* and *mod*, involved in restriction and modification, respectively. Both subunits are required for restriction, whereas Mod is sufficient to produce a modification. Finally, Type IV ‘restriction systems’, as opposed to R-M systems, are composed of one or two REases that cleave modified recognition sites (12).

R-M systems are major players in the co-evolutionary interaction between mobile genetic elements (MGEs) and their hosts. Closely related strains have different systems and distantly related species sometimes have similar systems, suggesting frequent HGT. This leads to weak phylogenetic association between systems and taxa (13–16), and typically one needs to compare strains within species to observe ortholog systems (17,18). Incoming DNA is unlikely to be modified in a way compatible with the R-M systems of the new host and will be degraded. This has led to very early proposals that R-M systems are bacterial innate immune systems (19), since they effectively allow self- from non-self discrimination. R-M systems might preferentially cluster with and stabilize other antivirus defense systems (toxin-antitoxin, abortive infection) in the so-called defense islands, i.e. discrete DNA segments that include a plethora of defense systems (5,20). In some cases, different defense systems have been shown to operate synergistically in order to increase the overall resistance to phage infection (21). Currently, it remains unclear, and is a matter of active research, the extent to which such co-localization occurs, its underlying mechanisms and if/how it translates into a functional cooperation between systems.

Some R-M systems can also propagate horizontally in a selfish way. Incoming DNA carrying an R-M system induces ‘genetic addiction’ to the host by post-segregational killing (22). This behavior leads to the stabilization of MGEs against challenge by competitor elements as long as the R-M system is present (23–25). Accordingly, genes encoding R-M systems have been reported to move between prokaryotic genomes within MGEs such as plasmids (23,26,27), prophages (27,28), insertion sequences/transposons (24,27), integrative conjugative elements (ICEs) (27,29) and integrons (27,30,31). In this regard, a mutual benefit is established between MGEs and R-M systems; the former facilitating horizontal transfer and the latter stabilizing it (31).

In other cases yet, the biological significance of some R-M systems remains obscure [reviewed in (32)]. For example, Type III systems are known to undergo phase variation (33) and Types I and II to affect the expression of certain genes (16,34), which might confer a fitness advantage to the host under certain environmental conditions. The processes underlying birth, death, pseudogenization (genetic degradation) or modification of the function of R-M systems also remain poorly understood, even though they are thought to be at the origin of ‘solitary’ (‘orphan’) REases and MTases (35,36), hybrid systems originated by the fusion of R-M components (37,38) or movement of DNA sequence recognition domains between different R-M systems (16,39,40).

The study of R-M systems is at a key point in time. On the one hand, a number of studies have enlarged the known scope of activity of these systems in bacterial cells (20,21,41), and a single resource, REBASE (42), has regrouped most of this information. On the other hand, the recent availability of tools to characterize bacterial methylomes is opening new perspectives on the effect of R-M systems in bacterial epigenetics (16,43). However, there is a lack of recent studies on some of the original questions put forward regarding R-M systems. How abundant are these systems? Which are more abundant? How rapidly do they evolve? How many systems are actually in MGEs? Which MGEs? Is there an association between R-M systems and different mechanisms of genetic mobility? In this work, we have used a comparative genomics approach to answer these questions. For this we have precisely identified MGEs and genes encoding mechanisms of transfer and protection against transfer. With such data at hand, we could characterize the associations between R-M systems and genetic mobility.

## MATERIALS AND METHODS

### Data

We analyzed 2393 chromosomes and 1813 plasmids representing 2261 fully sequenced prokaryotic genomes (2117 bacterial and 144 archaeal) and 831 complete phage genomes. These sequences and their annotations were retrieved from Genbank Refseq (ftp://ftp.ncbi.nih.gov/genomes, last accessed in February 2013). We used the definition of phages, chromosomes and plasmids of GenBank. We excluded genes indicated in the GenBank files as partial genes, as well as those lacking a stop codon or having one inside the reading frame. Curated reference protein sequences of Types I, II, IIC and III R-M systems and Type IV REases were downloaded from the data set ‘gold standards’ of REBASE (42) (last accessed in January 2013).

### Clustering analyses and construction of protein profiles

All-against-all searches were performed for REase and MTase standard protein sequences retrieved from REBASE using BLASTP (default settings, *e*-value <10^−3^), and the resulting *e*-values were log transformed and used for clustering into protein families by Markov Clustering v10–201 (44). To regulate the granularity of clustering, we have modified the inflation parameter (*I*) by increments of 0.2 in the range of 1.0 to 10.0 and proceeded with values of *I* = 1.2–1.4. In this process, we excluded proteins that were either redundant or very divergent in sequence length. Each protein family was aligned with MAFFT v7.0.17 (45) using the E-INS-i option, 1000 cycles of iterative refinement and offset 0. Alignments were visualized in SEAVIEW v4.4.0 (46) and manually trimmed to remove poorly aligned regions at the extremities. Hidden Markov Model (HMM) profiles were then built from each multiple sequence alignment using the hmmbuild program from the HMMER v3.0 suite (47) (default parameters). Type II MTases were retrieved using the PFAM-A profiles PF01555.12, PF02086.9, PF00145.1 and PF07669.5 (last accessed in February 2013). Types II and IV REases are very divergent and do not produce good multiple alignments (48), which precludes their use to build protein profiles. In these cases BLASTP was used to scan the genomes for homologs (default settings, *e*-value <10^−3^ and minimum coverage alignment of 50%). For Type IV REases and Type IIC systems, the control by co-localization with other genes of the system is not possible. To check the quality of our identifications, we compared them with the predictions of REBASE. For this, we sampled 10% of the replicons containing Type IIC systems or Type IV REases. Next, we queried REBASE for the total numbers of Type IIC systems and Type IV REases for each of these replicons. We have excluded REBASE hits corresponding to R-M systems interrupted by mobile elements or harboring any frameshifts. We found that for Type IIC systems our predictions and the ones from REBASE were practically identical (only 1.7% of the predicted Type IIC systems were not present in REBASE, whereas only 2.2% of REBASE predictions were lacking in our data set). For Type IV REases, 16.1% of our hits lacked in REBASE and 13.0% of REBASE predictions lacked in our list. We inquired on what would take to change our method to identify more REBASE predictions and found that if we do not require a minimum coverage of the alignment we could recover 98.4% of the REBASE predictions. Nevertheless, since some of these alignments are quite poor, we opted by keeping the coverage criterion at the cost of risking missing a small part of the systems.

### Identification of R-M systems and solitary R-M components

Types I, II and III R-M systems were identified by searching genes encoding the MTase and REase components at less than four genes apart. The output was subsequently curated in order to eliminate multiple occurrences of the same R-M system, for example as a result of the presence of two REase or MTase genes pertaining to the same R-M system. R-M systems containing more than one specificity (S) gene were considered as a single system. Situations involving ambiguous identifications may also occur, for example between REases of Types II and IV, or between Type IIC systems and other MTases or REases. In these cases, the R-M type was defined on the basis of the corresponding genomic context (presence or not of a linked REase or MTase) and on the output of the analysis of the system using REBASE. Type IIC R-M systems were defined as those including a gene encoding both a MTase and a REase function with similarity to Type IIC MTases and REases. An R-M system was defined as ‘complete’ if both REase and MTase were present. For the contextual analysis of R-M systems, we have considered two independent R-M systems as co-localized if their distance was below 10 genes. Genes of functionally linked components of R-M systems are typically co-localized, and although there are no apparent impediments for the existence of a functional link if the genes are distantly located in a genome (49), there are only very few distantly located R-M systems currently known to be functional (eventually as a result of biased searches), many of them being of Type I (40,50,51). We have considered a REase or MTase as ‘solitary’, if no cognate MTase or REase was found at a distance of less than 10 genes away, in a similar way to what was performed by others (35). At the current state of knowledge, one cannot use comparative genomics to infer a functional link between non-co-localized REase and MTase genes even though some such cases have been reported (36,52). Given the quick rate of R-M systems gain and loss described in this work, it is unlikely that many R-M systems could have their components encoded in distant regions in the genome.

### Analysis of substitution rates

All-against-all BLASTP searches were performed on the sets of putative R-M systems scanned in the genomes (default settings, *e*-value <10^−3^). Clustering was performed using the SILIX package v1.2.8 (http://lbbe.univ-lyon1.fr/SiLiX, last accessed in April 2013) (53) using a minimum identity threshold of 80% and default values for the remaining parameters. Singletons were eliminated from our data set. The remaining protein sequences (putative orthologs) were reverse-translated to the corresponding DNA sequences using PAL2NAL v14 (54). Pairwise rates of non-synonymous substitutions (dN), synonymous substitutions (dS) and ω (dN/dS) were computed using the yn00 program of the PAML package v4.4b (55) implementing the Yang and Nielsen method (56). Estimations yielding dS > 1 (corresponding to situations of substitution saturation and representing 16.1% of the total data) were discarded to improve the quality of estimation of ω.

### Identification and classification of prophages, conjugative elements, integrons and CRISPR-Cas systems

The identification and classification of prophages was performed as in (57). This corresponds to the genomes of temperate phages integrated in the bacterial chromosome and is therefore a data set different from the genomes of phages from GenBank, which were sequenced from virions and most often correspond to virulent phages. The identification of genes encoding the functions related to conjugation in ICEs and in integrative mobilizable elements (IMEs) was obtained as in (58). ICEs (also called conjugative transposons) encode the entire machinery required for conjugation between cells. IMEs encode relaxases but lack a complete conjugative transfer system, which is encoded in *trans* by another mobile element. These conjugative elements are very abundant in bacterial genomes (58). The presence of integrons was based on the simultaneous detection of tyrosine recombinases (PFAM family profile PF00589) and of the conserved specific region of integron integrases (a dedicated profile was built using HMMER) (59). Clustered regularly interspaced short palindromic repeats (CRISPRs) were identified following the methodology published in (60). Briefly CRISPRs were identified using the CRISPR Recognition Tool (61) using default parameters. For purposes of protospacer identification, (i.e. sequences from invading genetic elements that are incorporated into CRISPR loci after infection), BLASTN was used for similarity searches between CRISPR spacer sequences and R-M genes of complete systems (*n* = 7764) or R-M solitary genes (*n* = 6446) (default settings, *e*-value <10^−5^). Matches showing at least 90% of identity and less than 10% difference in sequence length between query and hit were retained. Clusters of *cas* genes were identified using MacSyFinder Abby S. S. *et al*., (submitted for publication, https://github.com/gem-pasteur/macsyfinder).

### Detection of competence systems

We gathered representative proteins pertaining to competence systems of experimentally studied Gram-positive and Gram-negative model systems, from which multiple alignments and HMM profiles were built. For Gram-positive bacteria, this was performed for the DNA-binding receptor ComEA, the cytoplasmic membrane protein ComEC, the adenosine triphosphate-binding protein ComFA, the traffic NTPase ComGA, the polytopic membrane protein ComGB, the major pseudopilin ComGC and the prepilin peptidase ComC. A PFAM profile was extracted for the DNA processing protein A DprA (PF02481.10). Protein profiles for Gram-negative bacteria were taken from the bacterial secretion system detection tool integrated in MacSyFinder. These include the outer-membrane/tip-located adhesin PilC, the prepilin peptidase PilD, inner-membrane pilus-associated protein PilM, pilot protein PilP, secretin PilQ, PilT/PilU ATPases, minor pilin PilV and major pilins PilA and PilE. PFAM profiles were extracted for the inner-membrane pilus-associated proteins PilN (PF05137.8) and PilO (PF04350.8). Loci encoding the natural transformation machinery were defined as having a maximum distance between two consecutive genes of five and a minimum number of six genes. On the basis of evidence gathered from the literature, we have considered the PilU (62) and ComC (63) components as facultative.

### Identification of core- and pan-genomes

We built core-genomes for each of the 43 species having at least seven complete genomes available in Genbank RefSeq (Supplementary Table S1) as in (64). A preliminary set of orthologs was identified as bidirectional-best-hits using end-gap free global alignment, between the proteome of a pivot and each of the other strain proteomes. Hits with less than 80% similarity in amino acid sequence or more than 20% difference in protein length were discarded. For every pairwise comparison, this list of orthologs was then refined taking into account the conservation of gene neighborhood. Because, (i) few genome rearrangements are observed at these short evolutionary distances (65) and (ii) HGT is frequent (2), genes outside conserved blocks of synteny are likely to be xenologs or paralogs. Hence, we combined the previously homology analysis with the classification of these genes as either syntenic or nonsyntenic, for positional orthology determination. Thus, positional orthologs were defined as bi-directional best hits adjacent to at least four other pairs of bi-directional best hits within a neighborhood of 10 genes (five upstream and five downstream). These parameters (four genes being less than half of the diameter of the neighborhood) allow retrieving orthologs on the edge of rearrangement breakpoints and therefore render the analysis robust to the presence of rearrangements. The core-genome of each species was defined as the intersection of pairwise lists of positional orthologs. It thus consists in the genes present in all genomes of a species and, therefore, can also be used to compare the genomic localization of homologous proteins between strains (see below). Pan-genomes are the full complement of genes in the species and were built by clustering homologous proteins into families for each of the 43 species. We determined the lists of putative homologs between pairs of genomes (including plasmids) with BLASTP and used the *e*-values (<10^−4^) to cluster them using SILIX. SILIX parameters were set such that a protein was homolog to another in a given family if the aligned part had at least 80% (stringent pan-genome) or 40% of identity (relaxed pan-genome) and if it included more than 80% of the smallest protein.

### Genomic location of chromosomal R-M systems

Integration regions containing a given R-M system were defined as the regions flanked by the two consecutive core genes that include the system (see above). These regions correspond to single/multiple integration and/or deletion events. They can be strain-specific (recent integration) or shared by different strains (ancestral acquisition). When these regions corresponded to rearrangement breakpoints, i.e. to cases where the flanking core genes in the focal genome were not consecutive core genes in at least another genome of the same clade, they were ignored and excluded from further analysis. We observed such cases for 13 out of 850 R-M systems (less than 2%) and 33 out of 1153 (less than 3%) solitary R-M genes.

## RESULTS

### Abundance and distribution of R-M systems in genomes

**Figure 1.**
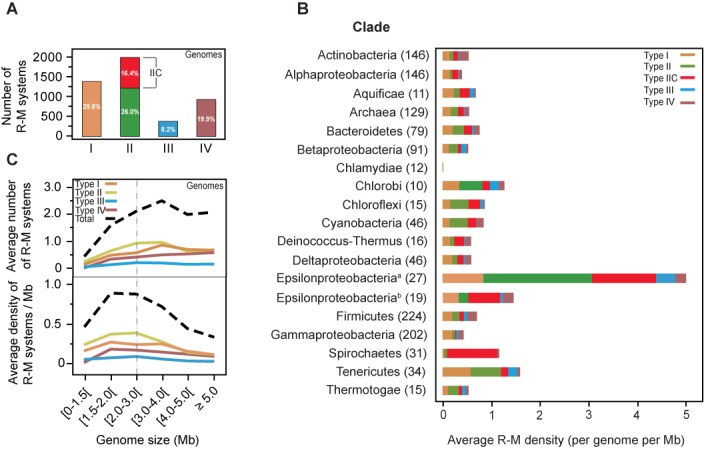
Quantification and distribution of R-M systems in 2261 prokaryotic genomes. (**A**) Amount of Types I, II, IIC, III R-M systems and Type IV REases found in genomes. Corresponding percentages are indicated. (**B**) Average R-M density (per genome per Mb) according to clade. The largest peak on R-M density observed for Epsilonproteobacteria (^a^) results from the presence of multiple systems particularly among *Helicobacter* species. For comparison, we also show the density for Epsilonproteobacteria without *Helicobacter* (^b^). Only clades with at least 10 different species were considered for comparison. The number of species within each clade is indicated next to its name. (**C**) Distribution of the average number of R-M systems per genome (upper graph) and average density per genome per Mb (bottom graph) according to genome size (Mb). Stippled line separates the regions having small and large genomes. Genomes of *Helicobacter* were not included to avoid obtaining extremely inflated values in the [1.5–2.2[ Mb genome size range.

We identified a total of 4743 R-M systems in 2261 prokaryotic genomes (Figure 1A and Supplementary Table S2). Type II systems are the most intensely studied and are also the most abundant (42.4%). Type IIC systems, in which the REase and MTase are part of the same polypeptide (Supplementary Figure S1), account for more than a third (38.8%) of all Type II R-M systems. Our results point to an average value of 0.54 Type II (excluding Type IIC) R-M systems per genome, which is considerably higher than the ratio of 0.35 previously found in the literature (35). Type I are the second most abundant, corresponding to ∼29.5% of all R-M systems. Type IV (methylation targeted) REases were found to be much more abundant (19.9%) than Type III (8.2%). We found similar trends in the relative amounts of R-M systems when the analysis was performed in chromosomes and plasmids separately (Supplementary Figure S2A and B). It should be noted that we used different appropriated methods to search for R-M systems. These include BLAST-based methods (REases of Type II and Type IV) and HMM-based methods (all other components). HMM-based methods are more sensitive (66), and we cannot exclude the possibility that we have under-estimated the number of Type IV REases (see the Materials and Methods section for comparisons with REBASE). This large number of Type IV REases is somewhat surprisingly, given the very few studies devoted to them.

The frequency of R-M systems varies widely among bacterial large phyla (Figure 1B; see also Supplementary Figure S2C for clades with less than 10 species in the genome data set). Some clades, such as Alphaproteobacteria or Chlamydiae, have very few systems even when controlled for genome size (less than one system per Mb). One could argue that the presence of many bacteria with small genomes rarely engaging in HGT would lead to fewer events of acquisition of R-M systems and weaker selection for these systems in these clades. However, this does not seem the case for Alphaproteobacteria, as this clade typically shows lower numbers and densities of R-M systems than the remaining Proteobacteria irrespectively of genome size (Supplementary Figure S3A). Hence, taxonomy and genome size may both be important variables determining the number of R-M systems in genomes. For Chlamydiae, the reduced size and number of genomes available does not allow to disentangle between the effects of the two variables. On the other extreme of R-M systems abundance, Epsilonproteobacteria contain by very far the highest number and density of R-M systems. While starting to analyze this question we met with previous observations showing that the genomes of *Helicobacter* harbor an exceptionally high number of R-M systems compared to other genera (average number of R-M systems in *Helicobacter* = 11.8 per genome) (16,32,67,68). We removed these genomes from further analyses because they have been studied before and indeed strongly inflate the statistics in the genome size range [1.5–2.2[ Mb. This reduced the size of the genome data set by only 2.3% to 2210 genomes. Tenericutes, which include wall-less bacteria such as *Mycoplasma*, show the second highest density of R-M systems. This is surprising, because this clade includes almost only genomes with sizes below 2 Mb (average genome size = 0.892 Mb). Recent reports show clear evidence of frequent horizontal transfer in this phylum (69,70), which might favor the acquisition of R-M systems. Overall, 74.2% of the genomes harbor R-M systems, and no clade is entirely devoid of them. R-M systems are therefore ubiquitous, but the patterns of their distribution are very diverse and depend on genome size, taxonomy and lifestyle.

R-M systems have been shown to be more abundant in larger genomes (20,32). We observed a positive correlation between the total number of R-M systems and genome size (Spearman's *ρ* = 0.2256, *P* < 10^−4^) (Figure 1C). However, this correlation is more complex than previously suggested. For small genomes (<2 Mb), there is a quick increase in the number (Spearman's *ρ* = 0.4758, *P* < 10^−4^) and density (Spearman's *ρ* = 0.3810, *P* < 10^−4^) of R-M systems with genome size. For larger genomes (≥2 Mb), the average number of R-M systems is nearly independent of genome size (Spearman's *ρ* = −0.0284, *P* > 0.2) and kept around two per genome. In other words, the number of R-M systems rises with genome size, but the effect saturates for genomes larger than 2 Mb. Accordingly, the density of R-M systems decreases with increasing genome size for this group (Spearman's *ρ* = −0.3434, *P* < 10^−4^). These correlations are qualitatively similar when including *Helicobacter* data (Supplementary Figure S3B) or when excluding the very abundant Type II systems from the analysis (Supplementary Figure S3C). Similar trends in the distribution of R-M systems were also observed when the analysis was performed in chromosomes and plasmids separately (Supplementary Figure S4).

### R-M systems are over-represented in naturally competent organisms

**Figure 2.**
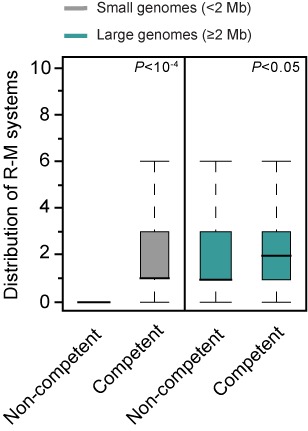
Box plots of the co-occurrence of R-M systems and natural competence machinery in small (<2 Mb) and large (≥2 Mb) genomes. Error bars represent standard deviations. Mann–Whitney–Wilcoxon test *P* value is indicated next to the box plots.

Horizontal transfer can take place by natural transformation which relies on a complex membrane machinery that, with few known exceptions, is closely related to Type IV pili and Type II secretion systems (71). *Helicobacter pylori* is competent and has many R-M systems, which has led to the suggestion that bacteria amenable to natural transformation have more R-M systems (32). However, *H. pylori* is one of the exceptions and uses a derivative of a Type IV secretion system for transformation (72). As mentioned above, it is also an outlier in the distribution of R-M systems. To test the statistical association between the ability for natural transformation and the number of R-M systems, we predicted the presence of the machinery of transformation from genome data. For this, we built protein profiles for the components of DNA uptake systems and used them to query the genomes of 904 Proteobacteria and 463 Firmicutes with MacSyFinder (see the Materials and Methods section for further details). The results of this analysis must be taken with care, since many bacteria encoding the full competence machinery have not yet been proven to be competent, presumably because the conditions where competence is expressed have not yet been found (73). Our results show that indeed genomes encoding the repertoire of genes involved in natural transformation have more R-M systems (*P* < 10^−4^; Mann–Whitney–Wilcoxon test). This result remains valid when we split the data set into small (<2 Mb) (*P* < 10^−4^; Mann–Whitney–Wilcoxon test) and large (≥2 Mb) (*P* < 0.05; Mann–Whitney–Wilcoxon test) genomes (Figure 2). These findings confirm the association between the presence of HGT and the abundance of R-M systems in genomes. It remains to be understood if naturally transformable bacteria over-represent R-M systems because these systems may be frequently acquired by natural transformation and stabilized by post-segregational killing or because these systems are particularly advantageous for naturally transformable bacteria.

### The genetic mobility of R-M systems

We showed that the abundance of R-M systems is associated with the existence of HGT even in naturally transformable bacteria where transfer does not require selfish MGEs. This raises the question of the role of R-M systems in MGEs, independently of their effect on HGT. R-M systems were discovered by their ability to prevent phage infection, but some phages also encode R-M systems (74). We searched for R-M systems in 831 complete phage genomes available from GenBank and only identified nine R-M systems (seven of Type II and two of Type IV) (Supplementary Figure S5A). Accordingly, the density of R-M systems in phages is lower than those found in any other type of replicon considered in our analysis (Supplementary Figure S5A). This suggests that R-M systems are rarely associated with phages and that these are rarely vectors of their horizontal transfer. To evaluate the association between the ecology of phages and R-M systems, we also studied the R-M systems present in prophages. These are temperate phages that we identified integrated in the chromosome (see the Materials and Methods section). Interestingly, the density of R-M systems in our data set of prophages was 8-fold higher than in the data set of GenBank phage sequences (most of which are virulent phages) (*P* < 10^−4^; Chi-square test) (Supplementary Figure S5A). This suggests that temperate phages leading to successful lysogens are more likely to encode R-M systems than virulent phages.

**Figure 3.**
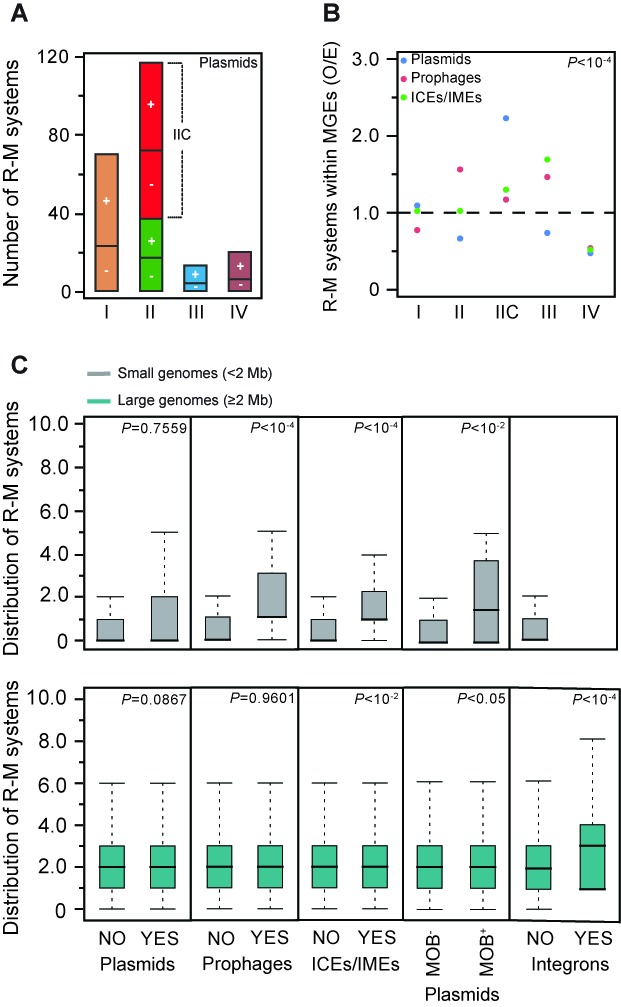
Quantification and distribution of R-M systems in MGEs. (**A**) Amount of Types I, II, IIC, III R-M systems and Type IV REases found in plasmids. The latter were classified according to their transmissibility: plasmids encoding the entire conjugation machinery or at least the relaxase (MOB^+^, shown as +), and plasmids lacking even the relaxase (MOB^−^, shown as −). (**B**) Observed/expected (O/E) ratios of R-M systems in plasmids, prophages and ICEs/IMEs. Expected values were obtained by multiplying the total number of each type of R-M system by the fraction of R-M systems assigned to each MGE. (**C**) Co-occurrence of R-M systems and MGEs. Box plots of the genomic co-occurrence of R-M systems with plasmids, prophages, ICEs/IMEs and integrons in small (<2 Mb) and large (≥2 Mb) genomes. Error bars represent standard deviations. Mann–Whitney–Wilcoxon test *P* values are indicated.

Several works have shown that R-M systems stabilize plasmids in cells by their addictive behavior (22,23,25,27). Surprisingly, we found very few systems in plasmids when compared to chromosomes (219 versus 3802). Plasmids are smaller and we do find that the density of R-M systems is around five times higher in plasmids than in chromosomes (*P* < 10^−4^; Chi-square test) (Supplementary Figure S5A). Nevertheless, only 10.5% of the plasmids encode R-M systems (Type IV REases included), whereas 69% of the chromosomes do so. More than half of the plasmids lack genes associated with conjugation (75). The rarity of R-M systems in plasmids might be the consequence of the presumably lower genetic mobility of these elements. To test this hypothesis, we divided the plasmids into two classes: plasmids encoding the conjugation machinery or at least the relaxase that allows them to be mobilized in *trans* by another conjugation machinery (MOB^+^, 44.6% from total), and plasmids lacking even the relaxase (MOB^−^, 55.4%). More MOB^+^ than MOB^−^ plasmids were found to contain R-M systems (respectively 113 versus 75, *P* < 10^−4^; Chi-square test) (Figure 3A). MOB^+^ plasmids also have higher density of R-M systems than MOB^−^, showing that larger replicon size is not enough to explain the higher abundance of R-M systems we observe in the former plasmids (respectively 2.89 and 2.23 per Mb, *P* < 10^−4^; Chi-square test). The abundance of R-M systems in plasmids is therefore linked with their ability to transfer horizontally by conjugation. Plasmids account for a very small percentage of all R-M systems even if they over-represent R-M systems relative to the size of their replicons.

We then aimed at identifying if some types of R-M systems were over- or under-represented in certain types of MGEs. For this, we computed the observed/expected (O/E) ratios of the number of each type of R-M system present within plasmids, prophages and ICEs/IMEs. In this test, the null statistical hypothesis is that the relative distribution of the types of R-M systems is similar in chromosomes and MGEs. The distribution of the different types of R-M systems in all these MGEs was different from that of the chromosome (all *P* < 10^−4^; Chi-square test) (Figure 3B). Type III systems appear as particularly over-represented in ICEs/IMEs (*P* < 10^−4^; Chi-square test). Type IV REases are under-represented in all MGEs, which is consistent with their role in defense against invading epigenetic DNA methylation systems (41). The most compact systems (Type IIC) are overabundant in all MGEs, and especially in plasmids. Accordingly, genomes known to harbor a very large number of plasmids (e.g. those from Spirochaetes) include many Type IIC systems (as seen before in Figure 1B). In fact, Type IIC systems found in Spirochaete plasmids comprise roughly 26% of all Type IIC systems detected in plasmids. In prophages, R-M systems are rare, but we still observed a significant over-representation of Type II systems. These systems are able to stabilize genetic elements by post-segregational killing and they could favor the stabilization of the lysogenic state as well as protect the host against infection by other phages.

Since MGEs appear to carry few R-M systems, we decided to quantify the association between the number of R-M systems and the presence/absence of the different MGEs in genomes. Large genomes (≥2 Mb) show no strong association between the number of R-M systems and the presence or absence of plasmids (independently of being MOB^−^ or MOB^+^), prophages and ICEs/IMEs (Figure 3C and Supplementary Figure S5B). Only integrons are more likely to be found in large genomes with more R-M systems. This latter observation is in agreement with previous works suggesting that R-M systems stabilize super-integrons (76). Among small genomes (<2 Mb), the number of R-M systems is positively correlated with the presence of prophages, ICEs/IMEs and MOB^+^ plasmids (Figure 3C). These results are consistent with the ones on the abundance of R-M systems in prokaryotic genomes, even when integron-, prophage- and plasmid-associated R-M systems are excluded (Supplementary Figure S5C). Overall, these data suggest that the abundance of R-M systems is indeed associated with genome size and the presence of MGEs because they are both associated with higher rates of horizontal transfer.

### Co-occurrence and co-localization of R-M systems

**Figure 4.**
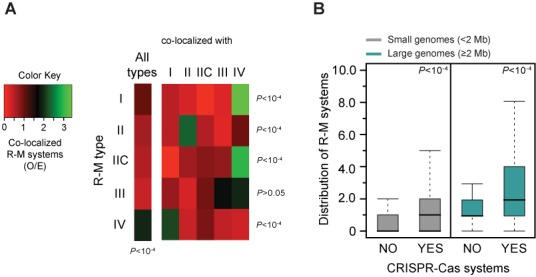
Associations between R-M and other defense systems. (**A**) Contextual analysis of R-M systems. Observed/expected (O/E) ratios of co-localized R-M systems (all types) and individual R-M types. Expected values were obtained by multiplying the total number of each type of R-M system by the fraction of R-M systems assigned to each MGE. (**B**) Box plots representing the co-occurrence of R-M and CRISPR-Cas systems in small (<2 Mb) and large (≥2 Mb) genomes. Error bars represent standard deviations. Mann–Whitney–Wilcoxon test *P* values are indicated.

The previous results suggest that bacteria enduring extensive HGT are more likely to encode R-M systems. We therefore analyzed the association of R-M systems with other systems dedicated to the control of MGEs. We started by analyzing the co-occurrence of the different types of R-M systems in genomes. We found significant co-occurrences for Type I R-M systems and Type IV REases (*P* < 10^−3^; Chi-square test), Types I and III (*P* < 10^−4^; Chi-square test) and Type IIC and Type IV REases (*P* < 10^−3^; Chi-square test). Co-occurrence could result from selection for a diversity of R-M systems in a genome or from the presence of the so-called defense islands (5,20). To test between these hypotheses we computed the number of R-M systems occurring at less than 10 genes apart in genomes. We found that only 10.0% of the R-M systems were this close in genomes (11.8% in genomes encoding at least two R-M systems). When we increased the neighborhood to 50 genes, the frequency of co-occurring systems only increased to 18.3%. Therefore, while we confirm previous observations of clustering of R-M systems in genomes (5,20), this affects a relatively small number of systems. We then analyzed systematically the pairs of close R-M systems (<10 genes apart). Type IV REases were found to often co-localize with other R-M systems (average inter-system distance = 3.2 ± 1.8 genes) (*P* < 10^−4^; Chi-square test) (Figure 4A). Co-localization was particularly striking with Type I systems (*P* < 10^−4^; Chi-square test) (Figure 4A), as previously observed (41). With the exceptions of Type IIC with Type IV, and Type II with itself, the remaining systems did not show significant co-localization patterns.

### Co-occurrence of R-M systems with other defense systems

CRISPR-Cas systems provide acquired immunity against viruses and other MGEs, being present in most archaeal (∼90%) and in a significant portion (∼40%) of the bacterial species for which genomes are available (77,78). CRISPRs are arrays of 24–28-bp direct repeats, separated by short unique sequences acquired from past infections (spacers) localized close to a cluster of *cas* genes, which form the basis for their specificity in the immune response. It has been recently reported that Type II CRISPR-Cas immune systems and Type II R-M systems work synergistically to prevent infection by phages (21). Indeed, we found that genomes encoding R-M systems are more likely to encode CRISPR-Cas systems (*P* < 10^−4^; Mann–Whitney–Wilcoxon test), for both large and small genomes (both *P* < 10^−4^; Mann–Whitney–Wilcoxon test) (Figure 4B). Many prokaryotes also encode homologs of the Argonaute (ARGO)-PIWI family of proteins, which have been recently found to be a bacterial defense system against MGEs (79,80). However, there is no significant co-occurrence of ARGOs and R-M systems in genomes (*P* > 0.05; Chi-square test, for both large and small genomes), even if ARGOs and CRISPR-Cas significantly co-occur (*P* < 10^−4^; Chi-square test). It should be noted that only 5.3% of all CRISPR-Cas systems co-occur in a proximity of ±25 genes of R-M systems and ARGO systems. The observation that co-occurrence of systems in the genomes is rarely associated with close co-localization in the genome suggests that co-occurrence is not caused by co-transfer, co-regulation in neighboring operons or co-occurrence in ‘defense islands’.

Since R-M systems are also involved in plasmid control, we hypothesized that CRISPR-Cas systems might target incoming R-M systems to prevent infection by plasmids. We tested this hypothesis by searching for sequence similarity between 80 685 CRISPR spacers identified in 1068 genomes containing such systems and our data set of R-M genes. We found only nine spacers (0.01% of the initial subset) with ≥90% coverage and identity with R-M systems (one single spacer with 100% identity). The same analysis performed with solitary REases and MTases resulted in only 14 (90% identity) and 9 (100% identity) spacers (see the Materials and Methods section and Supplementary Table S3 for details). These results show that R-M systems are rarely targeted by CRISPR spacers.

### Evolution of R-M systems

To gain insight into the evolutionary history of R-M systems, we built pan-genomes for a set of 43 bacterial species (Supplementary Table S1). Pan-genomes can be defined as the total gene repertoire of a set of strains of a given species, being composed of a core-genome harboring genes present in all strains, and an accessory (or dispensable) genome containing the remaining genes. We found only ∼4% of the R-M systems in the core-genome (Supplementary Figure S6). This tendency was common to all R-M types. The large majority of gene families (∼80%) are present in less than 1/3 of the strains (non-persistent genes), suggesting that they have been recently horizontally transferred.

**Figure 5.**
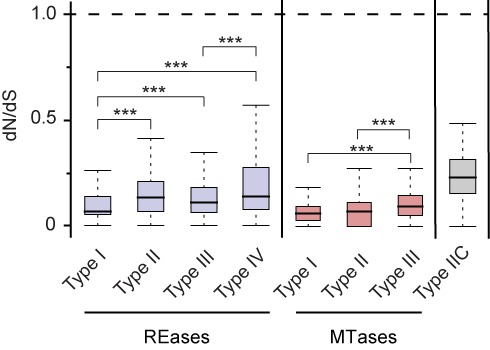
Evolution of R-M systems. Variation in dN/dS between REases, MTases and Type IIC systems. Error bars represent standard deviations. Significance was determined by computing Mann–Whitney–Wilcoxon test *P* values. For the sake of simplicity, we only show intra-REase and intra-MTase statistics. All remaining pairwise statistical comparisons are significant at *P* < 10^−3^ with the exception of Type I REases-Type III MTases (*P* < 0.05) and Type III REases-Type III MTases (*P* < 10^−2^). ***P < 10^−3^.

The rapid turnover of R-M systems in bacteria fits previous suggestions that R-M systems are rapidly lost because they are not selected for (35). To clarify this point in a population genetics setup, we inferred the pairwise non-synonymous (dN) and synonymous (dS) substitution rates as well as the dN/dS ratio for all REase and MTase genes of each type of R-M system. A dN/dS ratio exceeding 1 is expected if natural selection promotes diversification in the protein sequence (adaptive or diversifying selection), whereas a ratio below 1 is expected when the majority of non-synonymous changes are deleterious and systematically purged by natural selection (purifying selection). A dN/dS close to 1 suggests that most mutations are not under selection (neutrality). Our results show that both MTases and REases in all types of R-M systems are under strong purifying selection (dN/dS<<1; Figure 5 and Supplementary Figure S7). The observation of purifying selection on MTases is expected as long as the REase is active. Yet this is clear evidence against hypotheses that REases could evolve neutrally or under selection for inactivation once in prokaryotic genomes.

Not all enzymes evolve at similar rates (Figure 5). MTases show stronger purifying selection than REases, i.e. lower dN/dS, for all systems. This might explain why traditionally MTases are more conserved and easier to identify by sequence similarity (32). The dN/dS values of the different systems are also variable. We observed the highest dN/dS values for Types IIC and IV and the lowest for Type I. Interestingly, this suggests an association between the structural complexity of R-M systems and the intensity of purifying selection. Type IV and Type II proteins do not interact with other proteins and the former and Type IIC act alone, i.e. the entire system is composed of a single protein. The ensuing weaker structural constraints, the simpler co-evolution of restriction sites, and one single R-M protein might allow faster evolution. On the opposite extreme, Type I systems, which constitute the largest protein complexes and are thus presumably more structurally constrained, show the lowest dN/dS values.

### Solitary R-M genes

Previous works have found many solitary MTases and some solitary REases in genomes, suggesting they result from the genetic degradation of intact systems. Partial loss of R-M systems would lead to solitary MTases or REases that could eventually become domesticated by the host genome (35,36). Our observation confirms that components of solitary and complete systems are homologous, since we found more than 5000 solitary components in genomes using the same protein profiles and REBASE data that we used for the complete systems (Table 1). Yet demonstration of homology is not sufficient to suggest that solitary genes derive from complete systems. We have shown above that the vast majority of complete systems were not on the core-genome, suggesting they were very often acquired after the last speciation event. Therefore, to test the hypothesis that solitary components derive from complete systems one needs to show that this occurs at the level of the species. For this, we computed the number of protein families in the pan-genomes including complete R-M systems, solitary genes or both. If solitary genes often resulted from the degradation of complete systems, one would expect to find both types of elements in many pan-genome families. Instead, from the 525 (358) protein families of the pan-genomes encoding a complete R-M system (solitary component), only 9% (13%) also included a protein encoded by a solitary gene in another genome (note that sequencing and annotation errors will tend to inflate these numbers). Intriguingly, this observation suggests that most solitary R-M genes do not originate from the recent degradation of complete R-M systems.

**Table 1.** Numbers and densities (per element per Mb) of solitary MTases and REases found in chromosomes, plasmids, prophages and phages (temperate and virulent)

	# Solitary MTases (dens./element/Mb)	# Solitary REases (dens./element/Mb)
Chromosomes^a^ (2342)	3966 (0.593)	731 (0.116)
Plasmids (*n* = 1787)	292 (1.58)	23 (0.182)
Prophages (*n* = 2827)	717 (5.44)	15 (0.096)
Phages (*n* = 831)	143 (2.47)	12 (0.230)
Temperate phages (*n* = 311)	56 (3.55)	5 (0.290)
Virulent phages (*n* = 520)	87 (1.85)	7 (0.196)

^a^Results shown for chromosomes do not include R-M systems located in prophages.

Helicobacter genomes were not considered.

**Figure 6.**
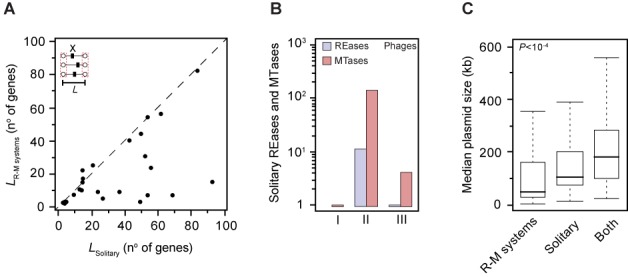
Comparative analysis of complete R-M systems and solitary components. (**A**) Median size of regions (*L*, expressed in the number of genes) harboring complete volatile R-M systems versus solitary volatile R-M elements (indicated as X). Stippled line corresponds to the identity. (**B**) The number of solitary REases and MTases in phages. Over 90% of the total hits were found to correspond to solitary MTases. (**C**) Median plasmid size (kb) for plasmids containing only complete R-M systems, solitary components or both. Mann–Whitney–Wilcoxon test *P* value is indicated next to the box plots.

If solitary R-M genes were derived from genetic degradation of R-M systems arising by horizontal transfer then they should be encoded in MGEs ongoing genetic degradation. Hence, MGEs carrying solitary systems should be smaller than those encoding complete R-M systems. To test this hypothesis, we computed the distance between the two flanking core genes surrounding solitary R-M genes and complete R-M systems. We found that for ∼80% of the species containing non-persistent R-M proteins, this distance was smaller for complete systems than for solitary proteins (*P* < 10^−2^; Binomial test) (Figure 6A). Accordingly, solitary elements are very abundant in large MGEs such as phages and conjugative elements, whereas we showed above that complete systems are rare in these elements. In phages we identified 155 solitary genes (Table 1 and Figure 6B), even though phages encode very few R-M systems. Plasmids containing only solitary R-M proteins are larger than those carrying complete R-M systems (median sizes of 106 and 50 kb, respectively, *P* < 10^−4^, Mann–Whitney–Wilcoxon test) (Figure 6C). These results strongly suggest that R-M systems and solitary proteins are transferred independently through distinct MGEs and/or transfer mechanisms. Additionally, this implies that solitary components of R-M systems are not systematically part of an ongoing genetic degradation process. Instead, it suggests solitary components are acquired as such by bacterial chromosomes.

To inquire on the nature of such adaptive functions, we compared the numbers of solitary REases and MTases. Solitary REases and MTases might be components of R-M systems encoded apart in the genome. Several of these have been found (35,36,81). In this case, one would expect a similar number of solitary REases and MTases. If solitary elements arose from random degradation of complete R-M systems, one would also expect to observe nearly as many solitary MTases as REases in the genome. On the other hand, solitary MTases and REases are known to have different impact in the cell fitness (36,52). Mutations leading to loss of the MTase while keeping the REase functional and expressed may be lethal if the restriction site is available in the genome. Accordingly, it has been shown that in bacteria solitary MTases are much more abundant than solitary REases (35). It is possible that the remaining REases are either associated with MTases in *trans* or are not expressed. Phages also show a strong predominance of solitary MTases over REases (143 against 12, *P* < 0.05, Chi-square test) (Table 1 and Figure 6B). Interestingly, phages over-represent solitary MTases (REases) four (two) times more than bacterial chromosomes (*P*<<10^−4^ and *P* < 10^−2^, respectively, Chi-square test). This suggests selection for the presence of solitary R-M genes in phages, and especially MTases. MTases with broad sequence specificities might be selected to avoid restriction by the host at the moment of infection. However, solitary MTases were found to be much more abundant in temperate than in virulent phages (*P* < 10^−3^; Chi-square test), and more abundant within prophages effectively present in the genomes than in the temperate phages of GenBank (*P* < 10^−2^; Chi-square test) (Table 1). This suggests that solitary MTases have some additional adaptive role in lysogeny.

## DISCUSSION

In line with many previous studies, we have shown here that R-M systems are nearly ubiquitous in Prokaryotes. We have also observed that their absolute abundance varies widely between phyla, within the limits of such an analysis using a database of bacterial genomes that is biased toward Proteobacteria and Firmicutes. Analysis of metagenomic data may allow in the future assessing if variations also occur between environments or clades that yet lack sequenced genomes. In the present data set, only the smallest genomes systematically lack R-M systems. These genomes typically correspond to sexually isolated endosymbiotic bacteria enduring very little or no HGT. This suggests that the link between genome size and HGT for small genomes is caused by the decreased frequency of HGT in many of the smaller genomes. Accordingly, the small genomes of bacteria that endure HGT, like the Tenericutes (including *Mycoplasma*), show high densities of R-M systems. Interestingly, several *Mycoplasma* encode R-M systems engaging in phase variation, which should increase their diversity (82). In the genome size range up to 2 Mb, one finds a variety of bacteria with increasing rates of horizontal transfer. Bacteria with genomes of ∼2 Mb like *Helicobacter*, *Haemophilus* or *Neisseria* are known to engage extensively in HGT. Larger genomes are expected to follow the same general trend within bacteria (2). For these genomes we did not observe a tendency of more R-M systems in larger genomes. Instead, the average number of R-M systems is kept approximately constant and equal to two systems per genome. R-M systems have been shown to decrease the probability of infection by naïve phages by values around 10^−5^ with strong variations from 10^−2^ to 10^−7^ (21,83,84). If the rate above is multiplicative, then two R-M systems will decrease the probability of phage infection by an average factor around 10^−10^. This is a simplified calculation assuming unbiased phage genome sequences (85) and lack of modification of the phage DNA (86,87), but under these conditions, additional R-M systems should provide little additional capacity of defense against MGEs. Moreover, they will increase the cost of genome methylation and the probability of accidental restriction of the chromosome. Hence, the presence of large numbers of R-M systems in some genomes likely requires additional explanations, for example the R-M addictive behavior and/or high rates of transfer of R-M systems.

Our work suggests that the distribution and evolution of R-M systems differs between types. We found a large number of Type IIC systems. To test if these might have arisen by fusion of head-to-tail-oriented REase and MTase genes in a single uninterrupted hybrid polypeptide we identified the Type IIC systems sharing medium to high similarity (>50%) with Type II systems encoded in two genes. Only 0.9% of the Type IIC systems have such level of similarity with other Type II systems (found mainly in Firmicutes), which suggests that such fusions are rare. Type IIC systems are more compact and their sequence specificity is linked in the same peptide with the REase and MTase functions. This might allow them to evolve new specificities faster (88), which is compatible with the observed rapid sequence evolution. Both rapid evolution and compactness might explain why they are more often encoded in MGEs. It is also possible that type IIC systems are more efficient at establishing in new hosts.

Some types of R-M systems co-occur with others more often than expected. Type IV REases are often encoded close to Type I R-M systems (Figure 4A). This conformation allows the degradation of unmethylated DNA recognized by the Type I and of modified DNA recognized by the Type IV system. Most Type I MTases methylate adenines to *N6*-methyladenines (m6A) (89). On the other hand, several known Type IV REases do not recognize m6A (12). The complementarity between the two systems might favor their clustering. It might also favor the evolution of broad substrate specificities in Type IV REases as long as this does not lead to the degradation of the DNA modified by the co-localized Type I system. The absence of selection for very specific sequence recognition might lead to more relaxed selection for Type IV REases. This is in agreement with the higher dN/dS ratios observed for these REases (Figure 5).

A number of works have shown that R-M systems can be stabilized in genomes and have an impact on the host genome composition (90–92). We observed relatively few R-M systems in plasmids, some in prophages, and practically none in phages. On the other hand, all these MGEs encode a large number of solitary R-M genes, notably MTases. While these results suggest that R-M systems may be used by MGEs to stabilize their presence in hosts, this occurs rarely in our data set. In contrast, we found that MGEs very often encode solitary MTases. These may serve as antidotes against R-M systems and thereby facilitate infection of new hosts and competition with other MGEs. Solitary MTases were suggested to result from complete R-M systems by loss of the gene encoding the REase (35). However, families of pan-genomes either include solitary genes or complete systems, but rarely both. Also, solitary MTases are typically transferred by larger MGEs than complete R-M systems. These results suggest that solitary genes arrive more frequently in genomes by HGT than by *in situ* genetic degradation of complete systems. Therefore, complete R-M systems and solitary R-M genes are largely independent sets of genes.

One intriguing question that remains to be clarified in more detail is how can R-M systems show such a high turnover in genomes when they are so poorly represented in MGEs? This question can be subdivided into two more specific ones: what is the source of the new R-M systems? Why are they so often lost? One possible explanation for the first question relies on the ability of certain R-M systems to behave as mobile units *per se*, sometimes generating extensive genomic rearrangements upon their insertion (27,93,94). Here we have observed that the majority of the acquired regions containing R-M systems are typically small (Figure 6A), suggesting that R-M mobility may be less dependent on MGEs and more dependent, for example, on the existence of small genomic integration hotspots. It is also possible that R-M systems frequently exploit other mechanisms such as natural transformation, vesicles, nanotubes, gene transfer agents or generalized transduction in order to move between genomes (73,95). This possibility is backed up by our data showing a higher number of R-M systems in competent than in non-competent bacterial hosts (Figure 2).

The frequent loss of R-M systems is apparently inconsistent with the observed strong selection against non-synonymous changes. These two observations may be reconciled if the selection pressure on the system fluctuates in time, i.e. if R-M systems alternate periods of strong purifying selection and periods of relaxed selection. The former would lead to the purge of non-synonymous changes and low dN/dS. The latter would lead to rapid gene loss. Relaxed selection might occur when there are many other R-M systems in the genome, especially if these have the same sequence specificity. In this case, there is competition between R-M systems resulting in relaxed selection for their maintenance in the genome. It could also occur when there is strong selection for HGT, e.g. in moments of stress, or when population cycles lead to the fixation of slightly deleterious changes, e.g. small population sizes or selective sweeps.

Our genome comparison analysis provides new insight into the intricate relationships between MGEs, R-M systems and other cell defensive systems. We found that such relationships are complex and depend on the type of R-M system and MGE involved. As a further intriguing novel feature, we observed that solitary R-M components and complete systems are essentially independent sets of genes. The growing access to bacterial methylome data will allow for a more comprehensive understanding of methylation specificity and how it affects bacteria genetic diversification and protection from MGEs.

## SUPPLEMENTARY DATA

Supplementary Data are available at NAR Online.

SUPPLEMENTARY DATA
