# Supplementary material for: The interplay of restriction-modification systems with mobile genetic elements and their prokaryotic hosts
Source: Nucleic Acids Res. 2014 Aug 12;42(16):10618–31. doi: 10.1093/nar/gku734 (PMC4176335; doi:10.1093/nar/gku734)
Supplement: SUPPLEMENTARY DATA [file supp_42_16_10618__index.html]

The interplay of restriction-modification systems with mobile genetic elements and their prokaryotic hosts — The interplay of restriction-modification systems with mobile genetic elements and their prokaryotic hosts — SUPPLEMENTARY DATA 

# The interplay of restriction-modification systems with mobile genetic elements and their prokaryotic hosts

## SUPPLEMENTARY DATA

**Files in this Data Supplement:**

- SUPPLEMENTARY DATA
- SUPPLEMENTARY DATA
