# Supplementary material for: The interplay of restriction-modification systems with mobile genetic elements and their prokaryotic hosts
Source: Nucleic Acids Res. 2014 Aug 12;42(16):10618–31. doi: 10.1093/nar/gku734 (PMC4176335; doi:10.1093/nar/gku734)
Supplement: SUPPLEMENTARY DATA [file supp_gku734_nar-01501-h-2014-File009.docx]

**The interplay of restriction modification systems with mobile genetic elements and their prokaryotic hosts**

Pedro H. Oliveira^1,2,^*, Marie Touchon^1,2^ and Eduardo P. C. Rocha^1,2^

^1^ Institut Pasteur, Microbial Evolutionary Genomics, Département Génomes et Génétique, Paris, France

^2^ CNRS, UMR3525, Paris, France

* **Corresponding author**:

Pedro H. Oliveira, Ph.D.

Institut Pasteur, Microbial Evolutionary Genomics, Département Génomes et Génétique, Paris, France

CNRS UMR3525

25 rue Dr Roux, 75724 Paris

Phone: 01 40 61 33 53

Fax: 01 45 68 87 27

E-mail: pcphco@gmail.com

**SUPPORTING MATERIAL**

**Nucleic Acids Research**

**Supplementary Figure 1**. Schematic representation of R-M gene organization. Type I systems are hetero-oligomeric proteins typically comprising two restriction (R), two modification (M) and one specificity (S) subunit composed of two target recognition domains (TRDs, shown as colored regions). The R subunit mediates DNA translocation and cleavage but is not essential for methylation. Type II systems are typically composed of homodimeric REases and monomeric MTases that possess separate TRDs and operate independently from each other. Type II REases are extremely varied and have the distinctive feature of cleaving DNA at a fixed position close to the corresponding recognition sequence. Type IIC enzymes are a particular subtype of Type II, which have both cleavage and modification domains within the same polypeptide. Variations in this case may be observed in the number of TRDs. Type III R-M systems consist of a restriction (*res*) subunit (responsible for DNA cleavage) and a modification (*mod*) subunit (involved in recognition and modification). Type III REases are either heterotrimers (Mod_2_Res) or heterotetramers (Mod_2_Res_2_), whereas the corresponding MTases have a homodimeric stoichiometry (Mod_2_). Type IV REases or ‘restriction systems’ are methylation-dependent enzymes with low sequence selectivity and a variable functional organization.

**Supplementary Figure 2**. Amount of Types I, II, IIC, III R-M systems and Type IV REases in 2,393 prokaryotic chromosomes (**A**) and 1,813 plasmids (**B**). The latter were classified according to their transmissibility: plasmids encoding the entire conjugation machinery or at least the relaxase (MOB^+^, shown as +), and plasmids lacking even the relaxase (MOB^-^, shown as -). (**C**) Average R-M density (per genome per Mb) in clades for which less than 10 different species were available. The number of species within each clade is indicated next to its name.

**Supplementary Figure 3**. Distribution of R-M systems in prokaryotes according to genome size. (**A**) Average number of R-M systems per genome (upper graph) and average density per genome per Mb (bottom graph) in Alphaproteobacteria and remaining Proteobacteria. (**B**) Effect of the presence/absence of *Helicobacter* genomes on the average number of R-M systems per genome (upper graph) and average density per genome per Mb (bottom graph). Both curves represent the cumulative of all types of R-M systems. (**C**) Effect of the presence/absence of Type II systems (Type IIC included) on the average number of R-M systems per genome (upper graph) and average density per genome per Mb (bottom graph).

**Supplementary Figure 4**. Distribution of R-M systems in prokaryotes according to replicon size. Distribution in the average number of R-M systems per replicon (upper graph) and average density per replicon per Mb (bottom graph) according to chromosome (**A, C**) or plasmid (**B, D**) size, with (**A, B**) or without (**C, D**) inclusion of *Helicobacter* genomes.

^a^ Results shown for chromosomes do not include R-M systems located in prophages

**Supplementary Figure 5**. Co-occurrence of R-M systems and MGEs. (**A**) Numbers and average densities (per element per Mb) of R-M systems found in chromosomes, plasmids, prophages and phages (temperate and virulent). (**B**) Box plots of the genomic co-occurrence of R-M systems with conjugative and mobilizable plasmids, ICEs, and IMEs for small (<2 Mb) and large (≥2 Mb) genomes. Error bars represent standard deviations. Mann-Whitney-Wilcoxon test *P* values are indicated next to box plots. (**C**) Distribution of R-M systems in prokaryotes according to replicon size in the absence/presence of R-M systems located in plasmids, prophages and integrons. Removing those R-M systems still leads to a positive correlation between number (Spearman’s ρ = 0.4808, *P*<10^-4^) and density (Spearman’s ρ = 0.3877, *P*<10^-4^) of R-M systems with size for small genomes. For larger genomes there is a weak negative correlation between the number of R-M systems and genome size (Spearman’s ρ = -0.0534, *P*<0.0281), and an inverse correlation between density of R-M systems and genome size (Spearman’s ρ = -0.3475, *P*<10^-4^).

**Supplementary Figure 6**. Frequency of R-M systems within the 43 pan-genomes (see also Supplementary Table 1). At the left-side extreme of the x-axis are the R-M systems present in single genomes, thus being regarded as strain-specific. At the opposite end of the axis are the R-M systems found in all genomes of a given species (strain frequency = 1). These are part of the corresponding core-genome.

**Supplementary Figure 7**. (**A**) Distribution of dN and dS values. (**B**) Evolution of R-M systems only for the 43 bacterial species used for construction of pan-genomes (see also Supplementary Table 1). Variation in dN/dS between REases, MTases and Type IIC systems. Error bars represent standard deviations. Significance was determined by computing Mann-Whitney-Wilcoxon test *P* values. For the sake of simplicity, we only show intra-REase and intra-MTase statistics. The remaining pairwise comparisons are significant at least at *P*<0.05 with the exception of Type II REases-Type III MTases, Type III REases-Type III MTases and Type IV REases-Type III MTases which are not significant. *** *P*<10^-3^, ** *P*<10^-2^, * *P*<0.05.

**Supplementary Table 1**. List of the 43 species with at least 7 complete genomes available for which we built core and pan-genomes.

**Supplementary Table 2 (See Excel file)**. Complete list of Type I, II, IIC and III R-M systems and Type IV REases detected in 2,261 prokaryotic genomes.

**Supplementary Table 3 (see below).** Complete list of CRISPR spacer sequences (and corresponding organisms in which they were detected), as well as R-M components (in both complete and solitary systems) to which the former share at least 90% identity and less than 10% difference in sequence length.

 **Footnote**: ‡ Multiple identity values correspond to multiple matches in different replicons.
